# Long-Term Effect of Diet Consistency on Mandibular Growth within Three Generations: A Longitudinal Cephalometric Study in Rats

**DOI:** 10.3390/biology12040568

**Published:** 2023-04-07

**Authors:** Ioannis A. Tsolakis, Christos Verikokos, William Papaioannou, Konstantina-Eleni Alexiou, Zafeiroula Yfanti, Despoina Perrea, Apostolos I. Tsolakis

**Affiliations:** 1 Department of Orthodontics, School of Dentistry, Aristotle University of Thessaloniki, 54154 Thessaloniki, Greece; 2 Department of Orthodontics, Case Western Reserve University School of Dental Medicine, Cleveland, OH 44106, USA; 3 Second Department of Surgery, “Laikon Hospital”, School of Medicine, National and Kapodistrian University of Athens, 10679 Athens, Greece; 4 Department of Preventive & Community Dentistry, School of Dentistry, National and Kapodistrian University of Athens, 10679 Athens, Greece; 5 Department of Oral Diagnosis & Radiology, School of Dentistry, National and Kapodistrian University of Athens, 10679 Athens, Greece; 6 Laboratory of Experimental Surgery and Surgical Research, School of Medicine, National and Kapodistrian University of Athens, 10679 Athens, Greece; 7 Department of Orthodontics, National and Kapodistrian University of Athens, School of Dentistry, 10679 Athens, Greece

**Keywords:** growth, soft diet, hard diet, mandible, condylar process

## Abstract

**Simple Summary:**

Craniofacial growth has been shown to be affected by different factors, including environment. It is thought that environmental changes could possibly affect the growth of the mandible. The question of how diet consistency affects mandibular growth within one generation of rats has been answered to some extent by various studies, according to which diet consistency may result in different masticatory forces that affect mandibular growth. There is no study so far that examined possible quantitative and qualitative growth changes in the mandible within different generations. The present experiment evaluated the impact of different food consistencies on mandibular growth within three generations. The results of this study indicate that a soft diet could be responsible for less mandibular growth, and this information might be passing through generations.

**Abstract:**

Background: This study investigated the effect of diet consistency on mandibular growth of Wistar rats through three generations. Methods: A total breeding sample of 60 female and 8 male Wistar rats were used in this study. Measurements took place only on female animals. Twenty female Wistar rats at 30 days old and four male rats at 30 days old comprised the primary breeding sample of the first generation, and from these animals two different generations were reproduced. Lateral cephalometric X-rays were taken from all female rats at the age of 100 days. A total of 7 craniofacial landmarks were selected for the linear measurements, and 12 curves and 90 landmarks were selected for geometric morphometric analysis of the lateral X-rays. Bonferroni test and a permutation test were performed for the statistical analysis. Results: Means of measurements of all soft diet groups compared to hard diet groups were significantly smaller. According to linear measurements, there was a significant difference only between the first-generation soft diet with the third-generation soft diet group. According to geometric morphometric analysis, the statistical differences appeared on the condylar process and the angle of the mandible. Conclusions: The soft diet could be responsible for less mandibular growth, and this information might be passing through generations.

## 1. Introduction

Hereditary and environmental factors are responsible for the growth of the craniofacial system [1,2]. It is well-accepted that muscular loading forces play their own part in bone growth and development [3]. One of the most critical muscular systems is the orofacial, which is necessary for feeding in vertebrates. Mandibular growth is closely associated with the movement of the jaws and loads of the orofacial region [4]. Mastication, as one of the environmental factors, seems to be responsible for a variety of developmental changes in the craniofacial region and more specifically in the mandible [5,6]. This may be the rationale for explaining the mean increase of malocclusions in industrial societies in the 20th century [7,8]. The current literature suggests that the shape of the maxilla, which deforms less in those with the ability to generate proportionately greater temporalis muscle forces, regulates the transmission of forces generated during mastication to the remainder of the skull [9]. Furthermore, current research shows a relationship between mandibular muscle force and form that is not as obvious from linear data. The best method for retrodicting masticatory muscle force and, consequently, mandibular loading is to consider the overall shape of the mandible [10]. It is hypothesized that the increased form variation exhibited in contemporary/urban populations is due to the lessening of functional limitations [11].

The primary factors that influence the facial growth are genetic and, to a lesser extent, functional and environmental factors. It is thought that force and function are closely related to one another. Genetic control, differential cellular responses, neurotrophic control, membrane control, oxygen tension, bioelectric potentials, pH levels, temperature effects, chalone-like inhibitors and stimulators, cyclic AMP, vasomotor control, nutrition, and enzymatic and hormonal factors are among the factors that influence the growth process. The distinction between primary and secondary or supporting elements is essential. Specific combinations of all the aforementioned factors, which regulate the face’s size, morphology, and position in the craniofacial skeleton, are likely to influence facial growth [12]. The human population in a modern civilization develops more severe malocclusions compared to populations from non-industrialized societies. Modern dietary habits have been identified as a factor in the increased frequency of malocclusion because they place less stress on the masticatory muscles than previous dietary habits. Therefore, the impact of diet consistency on craniofacial growth triggers the interest of researchers. Most of the research protocols looked over the effect of diet consistency to growth by using rats as experimental animals. Rats are usually the experimental animals of choice because they are small, can be easily housed, report a minimal social concern, have a short lifespan, and their genetic background and growth is well known, resembling those of humans [13,14,15,16,17,18,19,20,21,22,23].

The impact of genetics on craniofacial development as well as on occlusion is great and well established in recent years. Different family generations have been observed presenting similar craniofacial growth patterns. This led to the conclusion that craniofacial growth is heritable to some degree. It is also accepted that environmental factors can affect and change genetic information [24,25].

The coordinated actions of osteoclasts and osteoblasts result in the resorption and replacement of the existing cortical bone, a process known as intracortical bone remodeling (or simply remodeling). Secondary osteons, which are cylinder-shaped structures, are created as a result of this process. Due to their concentric lamellae and surrounding cement line, secondary osteons are apparent in cross-section. The remodeling process’ resorption phase releases mineral reserves to support mineral homeostasis, but it also leads to the development of microcracks as a result of mechanical deformation. Both significant mechanical deformations (high strain) and repeated cycles of loading have been linked to microcracks. Therefore, areas of the skeleton with more severe loading conditions should have increased rates of remodeling since those areas should sustain more microdamage. When the load situation is unknown, it is less evident if increased remodeling may be attributable to high strain or cyclical loading. The present literature includes controversial results according to the hypothesis that high strain may not be necessary for substantial remodeling to occur and that cyclical loading may be more likely to result in elevated remodeling [26,27,28,29,30,31,32].

Today, with the contribution of molecular biology, various epigenetic mechanisms are now known that consist of intracellular macromolecular chain reactions and extend from the membrane to the cell nucleus. In this way, information is transferred between the extracellular environment and the nucleus. The osteocyte network detects and responds to mechanical stimuli and thus plays an important role in triggering bone remodeling. In addition, loading applied to the tissues can change the shape of the cells. As a result, deformation of the intracellular content, including the cytoskeleton, is observed, and processes are activated that even change the mechanisms of action of the genome [33,34,35]. A period of 7 months with decreased masticatory needs in a soft diet group during adolescence and early adulthood resulted in smaller mandibles, as reported by Odman et al. in 2008. The area of the angular process and the inclination of the condylar process proved to have significant differences through morphometric analysis. This research used Sprague–Dawley rats as the experimental animal [36]. In 2007, the results of Tanaka et al.’s study on Wistar rats, it was revealed that the trabecular bone had a higher mineralization degree than the cortical bone. Higher mineralization levels appear in the anterior mandibular area than those in the posterior mandibular area. In those two areas, the soft diet group exhibited a higher degree of mineralization than the hard diet group. Increased mineralization was found on the trabecular bone in the condyle of the hard diet group than in the soft diet group [37]. Grunheid et al. in 2011 had a similar hypothesis but a different experimental animal (New Zealand white rabbits), and their results indicated that a moderate reduction in masticatory functional load does not drastically affect the remodeling rate [38]. In 2022, Tsolakis et al., by using Wistar rats as the experimental animal, found that a soft diet resulted in a smaller condyle and decreased angle of the mandible as well as the body of the mandible [39].

According to the present literature, it has been demonstrated that chewing hard food enhances nearly all physiological masticatory parameters, muscular coordination, and masticatory side modifications as compared to chewing soft food. These findings may help to explain the relationship between mastication and general health issues including obesity and decreased cognitive function as well as a more variable and symmetrical burden on the craniofacial structures that affects their growth and wellbeing [40].

The question of how diet consistency affects mandibular growth within one generation has been answered by various studies that had different results from that aspect. There are limited studies so far that looked for possible mandibular changes within different generations, and there is no similar study in rats.

The aim of this study is to examine the impact of different food consistencies on mandibular growth within one or even three generations.

## 2. Materials and Methods

An Institutional Review Board approval was obtained prior to the start of this study (General Directorate of Veterinary policy of the prefecture of the Attica of the Hellenic Republic. Approval Code: 1405). Subjects were recruited from the Laboratory of experimental surgery and surgical research, National and Kapodistrian University of Athens, school of Medicine, Athens, Greece. The study used an overall sample of 60 female Wistar rats and 8 male Wistar rats (Table 1). All measurements took place on female rats, and the males were used in this study only for reproductive reasons. For all measurements, the identity of the specimen was concealed during measurement. 

**Figure 1:**
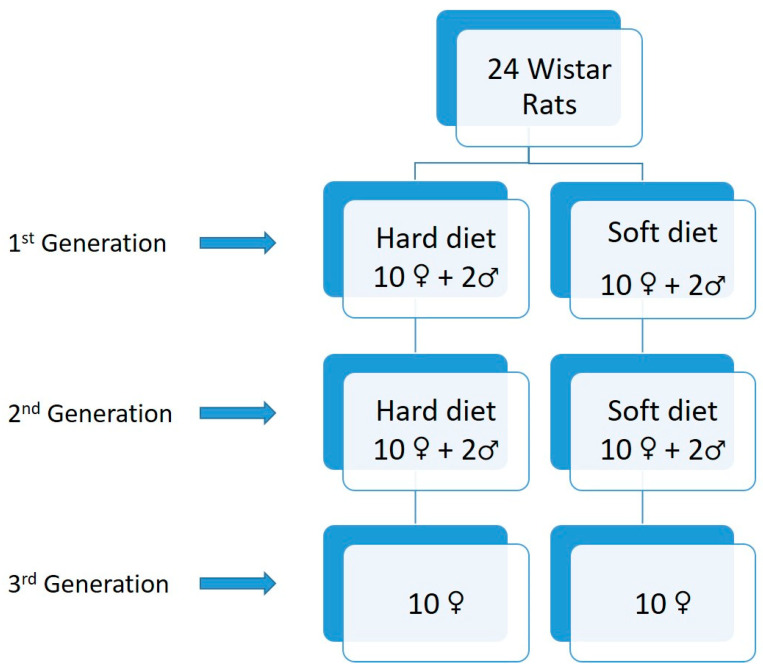
Diagram of the reproduction mechanism.

A total of 20 female Wistar rats at 30 days old and 4 male rats were the primary sample. The 20 females constituted the first generation. These were separated by computer-generated randomization into two equal groups of 10 females each. In the first group (S1), the rats were fed a soft diet while the second group (H1) was fed a hard diet, both for 30 days. Additionally, 2 males were fed with soft food, and 2 males of the same age were fed with hard food. On experimental day 31, female and male rats of each relevant group were mixed in order to reproduce. The female rats of the first generation were separated from their descendants after the ablactation period, and lateral cephalometric X-rays were taken (70th experimental day) before euthanasia. As a result, we had another two groups of the second generation, which were composed of 10 female rats randomly selected from the descendants of the first generation. The new groups were distinguished from the previous with new signs to those who were fed the soft diet (S2) and those fed the hard diet (H2). Additionally, 2 male rats from each descendant group of soft and hard diet of the first generation were kept in the experiment for reproduction purposes of the study. Forty-one days after, the female and male rats of each relevant group of the second generation were mixed in order to reproduce. The female rats of the second generation were separated from their descendants after the ablactation period, and lateral cephalometric X-rays were taken (150th experimental day) before euthanasia. From the descendants of the second generation, two groups of female rats were formed, composed of ten animals each randomly selected and representing the third generation. Thus, the third generation consisted of the soft diet group (S3) and the hard diet group (H3). Additionally, 2 male rats from each descendant group of soft and hard diet from the second generation were kept in the experiment for reproduction purposes of the study. Forty-one days after, female and male rats of each relevant group of the third generation were mixed in order to reproduce. The female rats of the third generation were separated and randomly selected from their descendants after the ablactation period and lateral cephalometric X-rays were taken (230th experimental day) before euthanasia. The reproduction mechanism is shown in Figure 1.

The regular rat diet was given to the hard diet groups as hard pellets (R34; Lactamin). The regular diet was crushed and combined in standardized amounts with water for the soft diet groups (2 parts food:5 parts water). The bedding material in the cages of this group was sifted to remove bulky items that would encourage excessive chewing.

**Figure 2:**
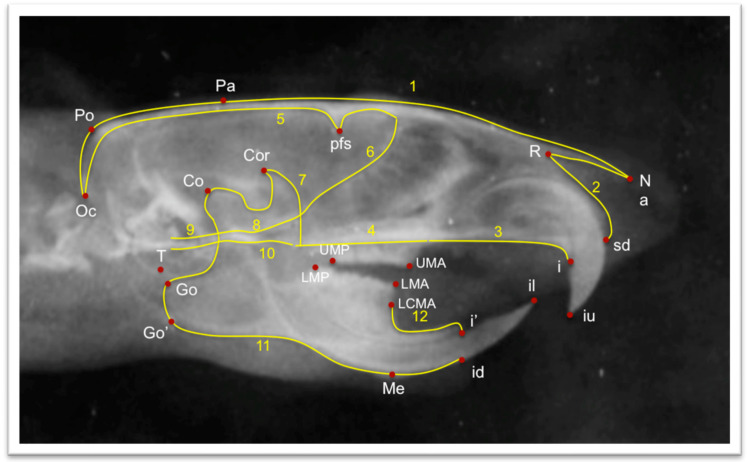
Traced lateral X-ray. The numbers correspond to the total number of the curves. The cephalometric landmarks correspond to Table 2.

According to the guidelines issued by Tsolakis et al., a unique cephalostat was created. The Carestream Insight No4 (3.1 × 4.4 cm) X-ray film (Atlanta, GA, USA) and the Siemens Nanodor1 X-ray machine (Erlangen, Germany)were both used [41]. The Siemens X-ray machine was set to 75 kilovolts and 15 milliamperes. For 0.8 s, the animals were exposed to radiation. To keep them stable throughout the procedure, all of the animals were given anesthesia. The X-ray films were manually developed, taking 5 min to develop and 8 min to repair. After fixation, the films were cleaned for an hour and then air-dried. The Epson V750 Pro (Los Alamitos, CA, USA) was used to scan the X-rays and convert them to digital format. Once the films were scanned, they were uploaded to the Viewbox software (Viewbox© version 4.1.0.10, dHAL software, Kifissia, Greece). The X-rays were traced after being uploaded. Seven landmarks were selected for the linear measurements of the lateral X-rays (Table 2). The linear measurements of choice were differentiated into those that measured the length of the mandible and those that measured the posterior height of the mandible (Table 3). For morphometric analysis, the major craniofacial structures shown on lateral cephalograms were digitally drawn using 12 curves and 90 landmarks, of which 74 were semi-landmarks and 16 were fixed landmarks. The first curve is composed of 14 semilandmarks, the second curve is composed of 3 semilandmarks, the third curve is composed of 3 semilandmarks, the fourth curve is composed of 3 semilandmarks, the fifth curve is composed of 11 semilandmarks, the sixth curve is composed of 6 semilandmarks, the seventh curve is composed of 4 semilandmarks, the eighth curve is composed of 4 semilandmarks, the ninth curve is composed of 3 semilandmarks, the tenth curve is composed of 3 semilandmarks, the eleventh curve is composed of 17 semilandmarks, and the twelfth curve is composed of 3 semilandmarks. The cranial base, maxilla, and mandible, as well as the entire craniofacial complex, were subjected to Procrustes superimposition and principal component analysis (PCA) to describe form variability. Once the X-rays were uploaded, the morphometric landmarks were digitized on the X-ray (Figure 2).

### Statistical Analysis

The operator’s reliability was calculated using intraclass correlation on 20 randomly selected subjects, whose data were re-measured 3 weeks apart.

For the linear measurements, regression analyses were used to evaluate differences that were related to diet and generation. The effects of diet, generation, and their interactions were regressed on each variable. Quantile regression was utilized when the residuals’ normality assumption failed. The Bonferroni method was used to correct comparisons for multiple comparisons. Analysis was performed at a statistical significance level of = 5%.

For the morphometric analysis, Procrustes ANOVA was used to generate values of *p* in order to establish whether shape differences were statistically significant. The Procrustes distance between the observers’ landmarked samples was tested for mean overall shape differences using a 10,000-round permutation test.

## 3. Results

This study investigated the influence of diet consistency on mandibular growth within one, two, and three generations. In the present study, twenty-four rats were grouped into a hard diet group and a soft diet group. After 30 days of growth, all rats reproduced. This procedure happened in order to produce three generations. We investigated the effects of dietary physical consistency on the mandibular growth, using lateral cephalometric X-ray. The sample included 60 female rats (Table 1). The intra-observer variations yielded an excellent agreement between all measurements. (Table 4).

### 3.1. Length Measurement

The linear measurements that indicated the differences on mandibular length were Go’-Me, Go-Me, Cor-Me, Co-Me, Co-id, Co-i’. As shown in Table 6, there were statistically significant differences between all soft diet groups when they were compared with the hard diet groups in all linear measurements. There were no statistically significant differences between all hard diet generations due to measurements. The linear measurements showed no statistically significant differences between the first generation and second generation of the soft diet groups, as well as between the second and third generations of the soft diet groups. There were statistically significant changes between the first generation and the third generation of the soft diet group in all measurements except Go’-Me (Table 5 and Table 6).

### 3.2. Posterior Height Measurement

The indications for the posterior mandibular height were Co-Go, Co-Go’ linear measurements. There were statistically significant differences between all soft diet groups when they were compared with the hard diet groups in all linear measurements. There were no statistically significant differences between all hard diet generations due to linear measurements. The measurements showed no statistically significant differences between the first generation and second generation of the soft diet groups, as well as between the second and third generations of the soft diet groups. There were statistically significant differences between the first generation and the third generation of the soft diet group in all measurements (Table 5 and Table 6).

### 3.3. Geometric Morphometric Analysis

**Figure 3:**
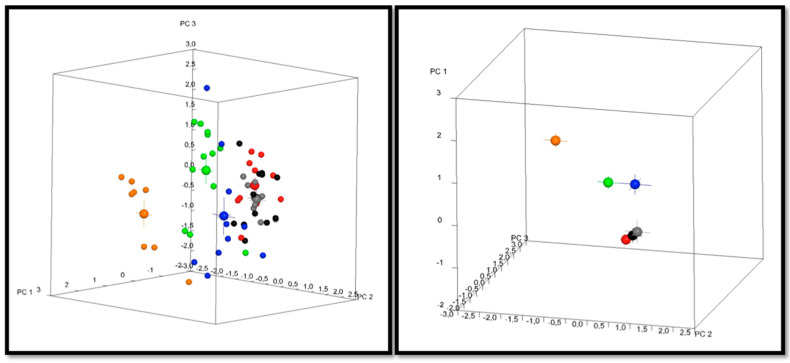
Plot sample for the morphometric analysis. S1 (blue), H1 (red), S2 (green), H2 (grey), S3 (orange), H3 (black). On the left box, all the measurements and their means are displayed. On the right box, the means of the measurements are displayed.

**Figure 4:**
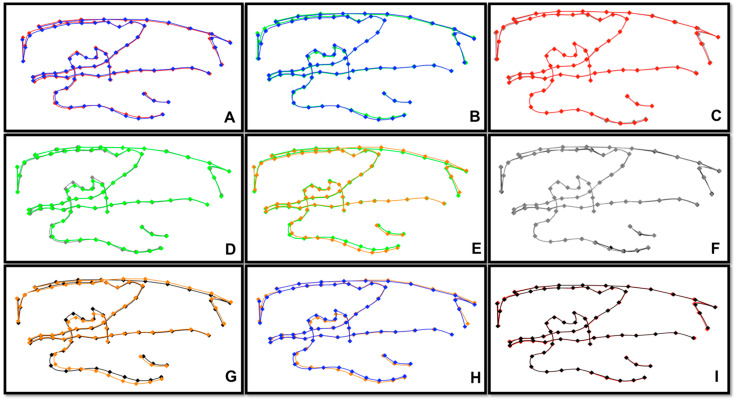
Corresponding superimposition of all means. (**A**) S1 vs. H1 (*p* = 0.002), (**B**) S1 vs. S2 (*p* = 0.048), (**C**) H1 vs. H2 (*p* = 0.054), (**D**) S2 vs. H2 (*p* = 0.001), (**E**) S2 vs. S3 (*p* = 0.001), (**F**) H2 vs. H3 (*p* = 0.41), (**G**) S3 vs. H3 (*p* = 0.001), (**H**) S1 vs. S3 (*p* = 0.01), (**I**) H1 vs. H3 (*p* = 0.083).

The first three principal components (PC1, PC2, PC3) accounted for 55.7% of the sample’s variability and described variability in the vertical direction, in the anteroposterior direction and in the gonial angle of the mandible, respectively. Significant differences between all soft and hard diet groups were revealed by the permutation test on the morphometric measures (*p < 0.05*). There were no statistically significant changes between the first, second, and third generations of the hard diet groups (*p > 0.05*). All comparisons between the soft diet groups revealed statistically significant differences. More specifically, the comparison of S1 and S2 groups resulted in a *p-value = 0.048*, the comparison of S1 and S3 resulted in a *p-value = 0.001*, and the comparison of S2 and S3 resulted in a *p-value = 0.01*. A regional Procrustes superimposition of the average shapes of Wistars was performed in order to visualize these differences. The superimposition of the morphometric means of SA and HA showed differences on the condyle, the angle of the mandible, and the body of the mandible. The superimposition of S1 and S2 means showed differences on the condyle, the coronoid, and the body of the mandible. The superimposition of S2 and S3 as well as the superimposition of S1 and S3 showed differences on the condyle, coronoid, gonial angle, and body of the mandible (Figure 3 and Figure 4). There were no shape differences between all groups on the cranium and maxilla. Hard diet rats had longer mandibular bodies, longer mandibular rami, posteriorly displaced coronoid processes, and shallow mandibular notches compared to soft diet groups, and there was no difference in shape between all hard diet groups. Soft diet rats exhibited shallower mandibular notches, shorter coronoid processes, and shorter condylar heads compared to the hard diet. These characteristics in soft diet groups were more exaggerated in the third generation.

## 4. Discussion

Previous studies have looked over the influence of diet consistency on growth using lateral cephalometric X-rays, but only one of them exceeded one-generation as the period of time. Our study showed statistically significant differences in height and length of the mandible. We found no statistically significant differences on the coronoid process of the mandible in the first generation; however, there were significant differences in the next generations. The differences showed that the coronoid process had a different height; it was displaced vertically, and it appeared smaller in size for the soft diet group. Kiliaridis et al. (1985) found that there was a significant difference in the size of the coronoid process, and it was smaller in the soft diet group [42]. However, in 2014, Hichijo et al. did not find any significant differences between the two groups [43].

In our study, a difference in the condyle between soft and hard diet groups was found in all three generations. The differences showed that the condyle was displaced vertically, and it was getting smaller in the soft diet group. In 1985, Kiliaridis et al. found linear differences on the condyle of the mandible between the two groups [42]. Two years later, Bouvier and Zimny (1987) agreed with this finding [44]. Maki et al. (2002) found that the height of the condylar process was increased in the hard diet groups [45]. In 2011, Dias et al. and Tiilikainen et al. found statistically significant differences between the soft and hard diet group on the condyle [1,46].

This present study found no significant differences in the gonial angle of the mandible in the first and second generations; however, there were significant differences in the third generation. Kiliaridis et al. (1985) found that the gonial angle was increased for the hard diet group [42]. In 2013, Guerreiro et al. showed that there were no statistically significant differences on the gonial angle between the two groups [47]. One year later, Hichijo et al. found that the gonial angle was decreased for the hard diet groups [43].

According to the present findings, the body of the mandible was found to be different in all three generations. The body of the mandible appeared to be displaced vertically. The length of the body did not change for the first and the second generation, but it changed on the third generation. In 1999, Killiaridis et al. found differences on the body of the mandible [48]. Abed et al. (2007) reported that the anterior corpus length and the ramus height were increased in the hard diet groups [49]. In 2014, Hichijo et al. found that there was no significant difference between the hard diet and soft diet groups in the mandibular length and the mandibular base length [43]. Recently, Tsolakis et al. found that there were significant differences in all measurements that correspond to the length and the posterior height of the mandible. There was a decrease in length and posterior height in the soft diet group. The morphometric superimposition revealed differences in the condyle, the angle of the mandible, and the body of the mandible [39].

It is important to mention that we used a similar methodology to previous studies, but with some of these studies, our results differ in terms of one-generation outcome. This could be possibly explained because we used a different rat strain than some of the previous studies. Another possible reason could be the limitations of 2D X-rays and the procedure that each study followed to take those. We used Tsolakis et al.’s procedure in order to be standardized [41]. Lastly, one possible reason for this could be that some studies did not examine the operators’ reliability in tracing the cephalometric X-rays.

There is only one study in the present literature that looked over the mandibular changes within different generations, but in this study, they used mice as an experimental animal. In 2020, Hassan et al. looked over the differences in craniofacial morphology between soft diet and hard diet groups within 15 generations of mice. According to their findings, short-term soft diet ingestion caused multiple morphological modifications as well as a considerable reduction in craniofacial size. However, shape analysis showed mice with shorter mandibles and crania in the anteroposterior dimension in their study. Long-term soft diet consumption over 15 consecutive generations was not linked to changes in craniofacial size. In addition, changes in shape lasted after diets were swapped for one generation, whereas size declined after one generation and subsequently reverted to baseline size, suggesting that changes in shape and size related to various functional loads appeared to be independent [50]. In contrast with this study, our study revealed that there were statistically significant differences between all soft diet groups when they were compared with the hard diet groups in all length measurements. Even though there were no statistically significant differences between the first generation and second generation of the soft diet groups, as well as between the second and third generations of the soft diet groups, the comparison of all length measurements between the first generation and the third generation of the soft diet group resulted in statistically significant changes. In addition, there were statistically significant differences between all soft diet groups when they were compared with the hard diet groups in all posterior height linear measurements. Furthermore, the posterior height measurements showed no statistically significant differences between the first generation and second generation of the soft diet groups, as well as between the second and third generations of the soft diet groups. Nonetheless, the comparison of all posterior height measurements between the first generation and the third generation of the soft diet groups resulted in statistically significant changes. Those results suggested that the long-term mastication of the soft diet was able to affect the morphology of the mandible, since there were differences between all generations, but the differences became statistically significant two generations later.

The craniofacial morphology, and more specifically the jaw morphology, affect mastication, speech, and breathing. It is important for scientists to understand how this can be altered and changed through life. According to the present study, diet can have a major impact on craniofacial morphology. The soft diet mastication could result in a more retrognathic mandible. It seems that there is genetic information passing through generations and most likely will appear to significantly affect the growth of the mandible after two generations. As a result of this, the mandible could grow more vertically. These results could suggest that the smaller mandible could be part of the human evolutionary craniofacial changes. According to the present literature, it is suggested that smaller mandible could lead to teeth crowding, lack of space for the third molars, and constricted airways. It is important to mention that the current literature has connected the less toned muscle activity of the craniofacial region and oropharynx to multiple chronic diseases such as cardiovascular problems, ADHD, and obstructive sleep apnea [51].

## 5. Conclusions

In conclusion, the findings of this study suggest that food consistency affects craniofacial growth and development. According to our findings, there are changes in the craniofacial development from the first generation of the soft diet group. The mandible appears to grow less in the soft diet group compared to the hard diet group. Means of measurements of all soft diet groups compared to the hard diet groups were statistically significantly smaller. According to linear measurements, there was a statistically significant difference only between the first-generation soft diet group compared to the third-generation soft diet group. According to geometric morphometric analysis, the statistical differences appeared in the condylar process and the angle of the mandible.

On the contrary, linear measurements and geometric morphometric analysis of hard diet groups of all three generations did not show statistically significant differences. It seems that the growth of the maxillofacial skeleton is affected by diet consistency, possibly passing through generations and instigating changes through different generations.

## Figures and Tables

**Table 1:** Sample size. S1 (the experimental group of the 1st generation that was fed with the soft diet), H1(the experimental group of the 1st generation that was fed with the hard diet), S2 (the experimental group of the 2nd generation that was fed with the soft diet), H2 (the experimental group of the 2nd generation that was fed with the hard diet), S3 (the experimental group of the 3rd generation that was fed with the soft diet), H3 (the experimental group of the 3rd generation that was fed with the hard diet).

	Gender	S1	H1	S2	H2	S3	H3
**Male**	8	2	2	2	2	0	0
**Female**	60	10	10	10	10	10	10
**Total**	68						

**Table 2:** Cephalometric landmarks.

Cephalometric Landmarks	Definition
Co	Most posterior-superior point on the mandibular condyle.
Go	Most posterior point of the angular process of the mandible
Go’	Point on the most inferior contour of the angular process of the mandible
Coronoid	Most posterosuperior point of condylar process
Me	The most inferior and anterior point of the lower border of the mandible
Id	Most inferior and anterior point on the alveolar process of the mandible
I’	The most anterior edge of the alveolar bone on the convexity of the lower incisor.

**Table 3:** Cephalometric measurements.

Structures	Cephalometric Measurements
Mandibular length	Co-Me
	Coronoid-Me
	Go-Me
	Go’-Me
	Co-Id
	Co-I’
Posterior mandibular height	Co-Go
	Co-Go’

**Table 4:** Reliability performed on 20 randomly selected subjects re-measured 3 weeks apart.

	Variables	Cochran’s Alpha
**Linear measurements**	Go’-Me	0.803
	Go-Me	0.827
	Coronoid-Me	0.828
	Co-Me	0.833
	Co-id	0.935
	Co-i’	0.929
	Co-Go	0.943
	Co-Go’	0.922

**Table 5:** Results for all variables measured, means, and standard deviations.

**Diet S**	**Generation**			
	**1** **(*n* = 10)**	**2** **(*n* = 10)**	**3** **(*n* = 10)**	**Overall**
	**Mean (SD)**	**Mean (SD)**	**Mean (SD)**	**Mean (SD)**
Go’-Menton	15.80 (1.86)	14.93 (1.40)	14.58 (0.72)	15.10 (1.46)
Go-Menton	17.38 (1.32)	16.77 (1.05)	15.79 (0.81)	16.65 (1.24)
Coronoid-Menton	13.98 (1.11)	13.47 (0.70)	12.65 (0.67)	13.37 (0.99)
Condylion-Menton	17.43 (1.24)	16.55 (0.67)	15.48 (0.90)	16.49 (1.23)
Condylion-Id	20.59 (1.17)	19.42 (0.87)	18.36 (0.93)	19.46 (1.34)
Condylion-I’	19.84 (1.16)	18.80 (0.97)	17.77 (0.98)	18.80 (1.32)
Condylion-Go	5.91 (0.49)	5.64 (0.64)	5.07 (0.63)	5.54 (0.67)
Condylion-Go’	6.97 (0.69)	6.45 (0.72)	5.95 (0.59)	6.46 (0.77)
**Diet** **H**	**Generation**			
	**1** **(*n* = 10)**	**2** **(*n* = 10)**	**3** **(*n* = 10)**	**Overall**
	**Mean (SD)**	**Mean (SD)**	**Mean (SD)**	**Mean (SD)**
Go’-Menton	18.65 (1.28)	19.44 (0.80)	18.85 (0.56)	18.98 (0.96)
Go-Menton	20.59 (1.42)	21.50 (1.01)	20.85 (0.71)	20.98 (1.12)
Coronoid-Menton	15.74 (1.42)	16.14 (0.29)	16.34 (0.40)	16.07 (0.87)
Condylion-Menton	20.74 (1.56)	20.69 (0.60)	20.27 (0.54)	20.57 (1.00)
Condylion-Id	24.47 (1.53)	24.60 (0.59)	24.40 (0.47)	24.49 (0.96)
Condylion-I’	23.42 (1.47)	23.69 (0.61)	23.49 (0.49)	23.53 (0.93)
Condylion-Go	7.65 (0.55)	7.10 (0.51)	6.77 (0.59)	7.17 (0.65)
Condylion-Go’	8.84 (0.61)	8.27 (0.46)	8.88 (0.74)	8.66 (0.71)

**Table 6:** Results for all variables measured for the linear measurements (*p* values). Differences related to diet and generation were assessed using regression analyses (bold font denotes a statistically significant difference).

	S1–H1	S2–H2	S3–H3	S1–S2	S1–S3	S2–S3	H1–H2	H1–H3	H2–H3
Go’-Me	***<0.001***	***<0.001***	***<0.001***	*0.437*	*0.105*	*>0.999*	*0.580*	*>0.999*	*>0.999*
Go-Me	***<0.001***	***<0.001***	***<0.001***	*0.858*	***0.007***	*0.194*	*0.266*	*>0.999*	*0.746*
Coronoid-Me	***0.001***	***<0.001***	***<0.001***	*0.891*	***0.002***	*0.067*	*>0.999*	*0.5789*	*>0.999*
Co-Me	***<0.001***	***<0.001***	***<0.001***	*0.208*	***0.004***	*0.076*	*>0.999*	*>0.999*	*>0.999*
Co-Id	***<0.001***	***<0.001***	***<0.001***	*0.092*	***0.003***	*0.853*	*>0.999*	*>0.999*	*>0.999*
Co-I’	***<0.001***	***<0.001***	***<0.001***	*0.151*	***0.006***	*0.877*	*>0.999*	*>0.999*	*>0.999*
Co-Go	***<0.001***	***<0.001***	***<0.001***	*>0.999*	***0.007***	*0.120*	*0.144*	*0.150*	*0.810*
Co-Go’	***<0.001***	***<0.001***	***<0.001***	*0.302*	***0.003***	*0.348*	*0.208*	*0.300*	*0.718*

## Data Availability

The corresponding author will provide the datasets used and/or analyzed during the current work upon reasonable request.
